# The pain, depression, disability pathway in those with low back pain: a moderation analysis of health locus of control

**DOI:** 10.2147/JPR.S139445

**Published:** 2017-09-29

**Authors:** Paul Campbell, Kate Hope, Kate M Dunn

**Affiliations:** 1 Arthritis Research UK Primary Care Centre, Institute for Primary Care and Health Sciences; 2 Keele Medical School, Keele University, Keele, Staffordshire, UK

**Keywords:** low back pain, health locus of control, moderation, depression, disability, Structural Equation Modeling

## Abstract

Low back pain (LBP) is common, impacts on the individual and society, and is a major health concern. Psychological consequences of LBP, such as depression, are significant barriers to recovery, but mechanisms for the development of depression are less well understood. One potential mechanism is the individual’s health locus of control (HLoC), that is, perception of the level of control an individual has over their health. The objective of this study is to investigate the moderation effect of HLoC on the pain–depression–disability pathway in those with LBP. The design is a nested cross-sectional analysis of two existing cohorts of patients (n=637) who had previously consulted their primary care physician about LBP. Measures were taken of HLoC, pain intensity and interference, depression, disability, and bothersomeness. Structural Equation Modeling analysis was applied to two path models that examined the pain to depression to disability pathway moderated by the HLoC constructs of Internality and Externality, respectively. Critical ratio (CR) difference tests were applied to the coefficients using pairwise comparisons. The results show that both models had an acceptable model fit and pathways were significant. CR tests indicated a significant moderation effect, with stronger pathway coefficients for depression for those who report low Internality (β 0.48), compared to those with high Internality (β 0.28). No moderation effects were found within the Externality model. HLoC Internality significantly moderates the pain–depression pathway in those with LBP, meaning that those who have a low perception of control report greater levels of depression. HLoC may signify depression among people with LBP, and could potentially be a target for intervention.

## Plain language summary

The experience of low back pain (LBP) includes various psychological consequences such as depression. Depression can be a significant barrier to recovery leading to increased disability; however, little is known on how depression develops. This study wished to test whether a person’s perception of control over their own health (health locus of control [HLoC]) could explain why depression develops. We carried out an analysis on over 600 people who had previously reported long-term LBP. We measured their level of pain and their depression levels and also their level of disability. We used a statistical model to see if HLoC levels changed the pain to depression to disability pathway. We found that people who have a low sense of control over their health reported more depression and that depression did associate with disability. This study helps to understand the link between pain, depression, and disability in those with LBP.

## Introduction

Low back pain (LBP) is common, affecting most people at some point in their lives. Lifetime prevalence varies from 40% to 80%, with a population point prevalence estimate of ~18%.^1^,^2^ Recurrence of LBP is also common; a review of cohort studies reports an estimated 70% recurrence rate over 5 years.^3^ LBP has a significant impact globally, comparable to conditions such as cancer, heart disease, and mental illness.^4^,^5^ This has considerable financial implications for medical care.^6^,^7^

A biopsychosocial approach has been applied to understand the etiology, assessment, and management of LBP.^8^,^9^ Within this approach, psychological factors are noted as important prognostic factors predictive of poor outcomes, they have a strong influence over pain management and coping, and as a result are key targets for intervention.^10^,^11^ One notable psychological factor is depression. Evidence shows that pain is a consistent risk factor for the development of depressive symptoms.^12^–^14^ Once in place, depression has been shown to be a significant barrier to recovery for those with LBP, and is a target for psychosocial pain management.^15^,^16^ However, the mechanisms that explain the development of depressive symptoms are less clear. Evidence shows that not all those who have LBP subsequently develop depression, and the factors associated with depression are many and varied.^17^–^19^ This evidence suggests potential latent or dispositional characteristics (ie, preexisting within the individual) that could moderate a person’s experience of pain and subsequent development of depression. One theoretical model,^20^ proposed to explain the development of affective disorders (eg, depression) from physical conditions (eg, pain), suggests that cognitive distortions, such as perceptions of helplessness and hopelessness associated with the experience of pain, can disrupt the day to day behaviors of the individual. Pain can affect social interaction (eg, reduced social networks and support), sleep (levels of disturbance), biological and neurological processes, and is associated with maladaptive coping strategies,^10^,^11^ all of which can result in increased distress and subsequent depression. A particular characteristic that fits well with the constructs of helplessness and hopelessness, within the context of illness or health condition, is a person’s health locus of control (HLoC).

A key feature of HLoC is the influence on perceptions of health.^21^ Based upon Social Learning Theory,^22^ HLoC is thought to be underpinned by personality traits, and is shaped through vicarious learning from significant others (eg, family members) and past experiences. Research has shown that HLoC is not a unidimensional construct but consists of two main components: Internal HLoC and External HLoC (hereafter termed Internality and Externality, respectively).^21^,^23^ Internality is a measure of the level of “self ” in determining an individual’s health and how much a person’s health is a consequence of their own behavior and actions. Externality is a measure of a person’s attribution of external influences on their health (eg, role of health care or doctor, luck, chance, or fate). Literature has shown that low levels of Internality (low estimation of self as agent for health) and high levels of Externality (high level of belief in external factors) within the individual can impact on a broad range of health conditions, such as coronary heart disease, dialysis management, and pulmonary conditions, as well as general morbidity, mostly in terms of low engagement for self-management.^24^–^27^ A recent meta-analysis of 76 independent studies illustrated the relevance of HLoC on a broad range of health outcomes and health behaviors.^28^ Furthermore, specific research evidence on HLoC and pain conditions has shown that HLoC constructs of Internality (ie, low) and Externality (ie, high) are associated with higher pain intensity, increased psychological morbidity in those with pain,^29^,^30^ and can influence an individual’s response to treatment.^31^,^32^ At present, pain research has considered HLoC as a covariate factor (ie, mediator explaining a relationship between pain and another variable, or associating with outcomes for those with pain), often focused on response to treatment. However, the principle of HLoC as a dispositional characteristic suggests a moderator role (ie, a condition on which an event occurs). Such information on potential moderators can enable the identification of subgroups of individuals at greater risk of poor outcomes who may then benefit from different or alternative treatments (eg, stratified care approaches).

**Figure 1:**
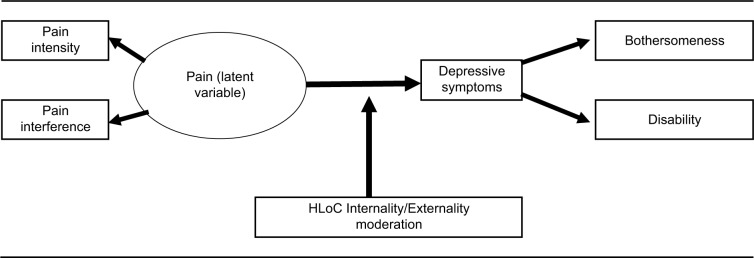
Path model of pain to depressive symptoms to disability and bothersomeness moderated by HLoC. **Abbreviation:** HLoC, health locus of control.

In line with the theoretical model of Cohen and Rodriguez,^20^ we wish to test the moderating influence of HLoC on the pain to depression to disability pathway in those with LBP. Within the model we wish to include a measure of pain interference with pain intensity, as a measure of the impact of pain, and also a measure of bothersomeness with disability, as an outcome to capture the subjective impact (severity) of LBP. We hypothesize that this pathway will be moderated by HLoC, with stronger associations between pain severity/pain interference, depressive symptoms, and disability/bothersomeness for individuals who report low levels of Internality and high levels of Externality, compared to individuals who report the reverse (Figure 1).

## Methods

### Design

This was a cross-sectional study of participants who had taken part in two longitudinal cohort studies.^33^,^34^ Ethical approval for the research studies was given by North Staffordshire and North West Cheshire Research Ethics Committees, respectively. All patients gave informed consent for participation, and all patients received usual care.

### Recruitment

Data for this current study are derived from two longitudinal cohort studies of primary care patients.^33^,^34^ Both cohorts had an initial baseline and follow-up for 12 months, and were then contacted and invited to take part at a second stage a number of years after. It is this later stage that forms the population for this current study. The first cohort study titled “Back Pain Research in North Staffordshire” (BaRNS) investigated a cohort of patients with LBP who presented at one of five general practices in North Staffordshire, UK.^33^ Participants aged 30–59 years who consulted with LBP during October 2001–October 2002 were mailed baseline questionnaires, and sent monthly follow-up questionnaires.^33^ The BaRNS cohort was then followed up again, 7 years later, with the primary aim of investigating long-term trajectories of those with LBP.^35^ In total, 338 participants were eligible to be contacted and 208 responded indicating a 62% response rate. It is this cohort (n=208), at this long-term follow-up point, who are included in this current study. The second cohort study “Beliefs about Back Pain” (BeBack) followed a similar recruitment methodology to the BaRNS study on a similar consulting population, using similar measures.^34^ Patients (aged between 18 and 60 years) who had consulted about LBP with their general practitioner (GP) were mailed baseline questionnaires and subsequent follow-up questionnaires.^34^ Recruitment for BeBack was carried out in eight primary care practices in North Staffordshire and Central Cheshire, UK in 2005–2006. This BeBack cohort was then contacted 5 years after their initial participation. In total, 696 of the original BeBack study participants were eligible for further contact at the 5 year stage, and 429 participants provided data for use in this study, indicating a 62% response rate.^36^ In total, by combining the cohorts, 637 participants were included in this current study. In both cohort studies, patients were identified by a Read Code classification of nonspecific LBP entered by their GP at the time the patient presented for consultation. Read Codes form the basis of classification of computerized patient records in UK general practices and validity has been established in the use of Read Codes for epidemiological research.^37^

### Measures

Measures used within this analysis were the same within both cohorts. Depressive symptoms were quantified using the Hospital Anxiety and Depression Scale-Depression (HADS-D).^38^ The depression scale (HADS-D) contains seven questions, and gives a scale range of 0–21 for depression (Cronbach’s alpha in this current study, 0.86); use and validation in back pain populations have been demonstrated previously.^33^,^34^,^39^ The predictor variables within the model were pain intensity and pain interference related to LBP, chosen to represent the actual pain intensity and the impact of pain intensity. Pain intensity was measured using the mean of three numerical rating scales (range 0–10, Cronbach’s alpha 0.93 in this current study) based on the participants’ lowest, usual pain intensity (during the prior 2 weeks), and current pain intensity.^33^,^40^ The measurement of pain intensity using a numerical rating scale is well validated with the use of combination scales (eg, least pain, usual pain, current pain giving greater accuracy in assessment).^41^ The measure of pain interference, taken from the validated SF-12 general health questionnaire,^42^ asks participants “During the past 4 weeks how much did pain interfere with your normal work (including both work outside the home and homework)” scored on a 0–10 numerical rating scale; this measure has been used in similar pain cohorts previously.^33^,^34^,^43^ Disability resulting from back pain symptoms was assessed using the validated Roland–Morris Disability Questionnaire (RMDQ).^44^ The RMDQ is a 24-point self-reported disability questionnaire which asks individuals to assess their level of disability “today,” with a higher score indicating a greater level of disability (range 0–24, Cronbach’s alpha 0.94 in this current study).^45^ In addition to the measure of disability, and in line with the Cohen and Rodriguez model,^20^ we also included a measure of behavioral disruption (ie, a measure over and above the assessment of impact on activities) using a question on the bothersomeness of pain. Bothersomeness about pain was measured using a single item question “Overall, how bothersome has your pain been in the last 2 weeks” with five response options: not at all, slightly, moderately, very much, extremely; this measure has demonstrated validity in LBP populations, both as a prognostic factor and as an outcome (as used in this current study).^40^

The HLoC scale was used to assess both Internal and External HLoC dimensions, and to test the moderation effect of the model.^21^,^23^ There are five questions on Internality and six questions for Externality. Both dimensions use a 6-point Likert scale. Based on recommendations from the original authors, and previous research, quartile scores (upper and lower) for Internality and Externality were considered as the cutoff points for the moderation comparison analysis.^21^,^46^,^47^ Cronbach’s alpha testing of reliability indicated 0.72 for Externality and 0.63 for Internality scales in this current study.

### Statistical analysis

Preliminary analysis was carried out in SPSS version 21 (IBM Corporation, Armonk, NY, USA). Data were screened for normality distributions (skewness, kurtosis) and showed all variables were within acceptable limits (skew value >2, kurtosis value >7).^48^ However, within the Structural Equation Modeling (SEM) path analysis subgroups, created by use of upper and lower quartile scores for Internality and Externality, data showed some multivariate non-normality. To account for this, corrected boot strapping using 1,000 samples was employed to generate estimates of the sampling distribution, which enables examination of parameter distributions that are not affected by non-normality.^49^ Missing data analysis was carried out and 2.6% of the values were missing in the dataset. Inspection by variable showed that Externality had missing data above 5% (actual 6.6%). A check of patterns of missing data, using Little’s missing completely at random test,^50^ showed that data missing from this variable were not missing completely at random. Further inspection showed that respondents who missed values for this variable were significantly older (mean 58.6 vs 55.6 years), but no other difference was found for any other variable (gender, pain intensity, disability, pain interference, bothersomeness, depression). Given that missing data levels were low and specific to age only, full information maximum likelihood estimation was applied to account for missing data within both the HLoC models. Descriptive statistics were carried out on all variables describing mean, standard deviation, median, and interquartile range for continuous data, and percentage proportions for categorical data where appropriate.

Path analysis was applied using SEM (AMOS version 21; IBM Corporation) to test the moderation effect of HLoC. The influence of pain on depression was tested by creating a latent exogenous predictor variable combining the measure of pain intensity and pain interference on the predictive pathway to depressive symptoms. The latent variable was constructed to simulate both the actual level of pain intensity as well the impact of pain intensity in terms of the interference this would have on the person with LBP. Depressive symptoms variable was then set as the endogenous predictor of both disability (RMDQ) and bothersomeness. Bothersomeness was chosen because this may represent a subjective evaluation of pain via depression, in comparison to a more objective measurement of disability and function (RMDQ). Bothersomeness has been used as an independent outcome previously and has been shown to be independently associated with both pain intensity and disability^40^ (see Figure 1 for path model).

An a priori decision was made to examine the modification indices of each model to indicate options to improve model fit, as testing of initial models often results in poor fit due to miss-specified parameters.^51^ Modifications were considered only for error covariance (ie, not modifying pathways), based on the assumption that variables may share distinct covariance with each other, over and above their relationships within the model. Two models were then created using the same structure specifications, one for Internality (upper and lower quartile, n=302) and one for Externality (upper and lower quartile, n=305). Sample size calculation based on expected power of 0.8, and on expected direct effects (0.25–0.45) for SEM, indicates 140–300 participants for model stability.^52^

Recommendations for the assessment of model fit,^51^,^53^,^54^ suggested three fit indices; confirmatory fit index (CFI; >0.90 acceptable; >0.95 excellent), goodness of fit index (GFI; >0.90 acceptable; >0.95 excellent), root mean square error approximation (RMSEA; >0.1 poor; <0.08 acceptable; <0.05 excellent). Each model (Internality, Externality) was tested and evaluated for model fit, and then within each model simultaneous analysis was carried out to ascertain the comparative effect of HLoC moderation (eg, for Internality; low Internality vs high Internality, for Externality; low Externality vs high Externality). All models were adjusted for age and gender. Critical ratios (CRs) tests were applied simultaneously to each HLoC model. CR testing is an extension of SEM using a multigroup analysis function where mean values of observed variables are derived from the covariance matrix and compared using Z ratio tests.^55^ In this current model, CRs were examined to indicate significant moderated differences (CR score ≥1.96 indicates significant difference between comparison groups) within the parameters,^55^ indicating where potential moderation effects are within each model following previous methodology.^56^ Standardized regression coefficients for each pathway are reported with bootstrapped bias corrected 95% confidence intervals (CIs).

## Results

Participant characteristics are presented in Table 1. The mean age of participants was 55.8 years with the majority being female (62.3%). The mean scores for the outcome measures are also shown in Table 1.

### Model testing

Initial model fit was poor for both the Internality and Externality models (CFI <0.5, GFI <0.6, RMSEA >0.1). Examination of the modification indices in both models showed that allowing shared error covariance of depressive symptoms with disability and bothersomeness would improve model fit. A decision was taken to proceed with this modification based on the inference that there may be mood effects that relate to both disability and bothersomeness that are not influenced solely by pain or pain interference (previous literature has shown independent associations for these variables^40^). These modified models provided a good fit to the data: Internality model; CFI =0.97, GFI =0.96, RMSEA 0.067 (90% CI 0.043–0.091), Externality model; CFI =0.97, GFI =0.96, RMSEA 0.068 (90% CI 0.044–0.091).

### Moderation effect of HLoC Internality

All pathways within the models (low Internality and high Internality) were statistically significant, indicating support for the pathway. Pain and pain interference (latent variable) significantly predicted depressive symptoms, which in turn significantly predicted disability and bothersomeness. Results using standardized estimates are shown in Table 2, for both low Internality and high Internality groups. Examination of the CR revealed a significant moderation effect of Internality on the pain/pain interference pathway to depressive symptoms (CR 2.41, *p*<0.05). Pathways from depressive symptoms to disability (CR 1.46, *p*>0.05) and bothersomeness (CR 1.70, *p*>0.05) were not significantly moderated. Inspection of the standardized beta for the pain to depressive symptom pathway (ie, pathway with significant CR test) showed a significantly stronger effect for those with low Internality (β 0.48; 95% CI 0.35–0.62) compared to those who reported high levels of Internality (β 0.28; 95% CI 0.18–0.40).

### Moderation effect of HLoC Externality

As with the findings on Internality, all pathways in the Externality model for both groups (low Externality and high Externality) are statistically significant, supporting the overall structure of the model (ie, pain to depressive symptoms to disability and bothersomeness). Standardized estimates of the pathways are shown in Table 3 for both low Externality and high Externality groups. Inspections of the CRs show no significant moderation effect of Externality on the pathway model, and inspections of the standardized beta values showed similar coefficients between groups.

## Discussion

This is the first study to test the moderation effect of HLoC on the development of depression in those with pain within a path analysis model. The results show partial support to the study’s hypothesis that those who have low Internality and those with high Externality would report a greater strength of association between pain/pain interference and depressive symptoms and disability/bothersomeness. Significant moderation was found for the effect of Internality on the pain/pain interference to depression pathway, with almost doubling of the strength of association for those with low Internality compared to those with high Internality. However, the subsequent pathway from depressive symptoms to disability and bothersomeness was not moderated for Internality, and no moderation effect was found for Externality for any of the model pathways. This suggests that HLoC Internality may moderate the development of depression in those with pain and pain interference; however, it has less of a moderating effect in the role of depression to disability.

### Comparison with existing literature

Overall, the structure of both models was supported with good fit for a model where pain and pain interference predict depression which in turn predicts disability and bothersomeness. Certainly, the effect of pain intensity associating with depression, and depression associating with disability is not new, and the associations found in this study have support within the literature.^12^,^14^,^18^,^33^,^40^ For the findings of HLoC, research in other health conditions has shown that lower levels of Internality and higher levels of Externality are associated with poorer outcomes for those with heart disease,^24^ multimorbidity,^27^ epilepsy,^57^ and pulmonary complications,^26^ as well as self-rated health outcomes in the general population.^28^,^58^ While no previous research has considered moderation path analysis of HLoC on the outcomes for those with LBP, focusing on pain research, the picture is similar in terms of the associations of HLoC to pain-related outcomes. For example, one study of patients with LBP not only reports expected associations between pain, depression, and disability (ie, associations in similar direction to this model) but also shows independent associations of HLoC Internality with pain and disability, which gives some indication of potential independent influences of HLoC Internality.^59^ Similarly in another study, a greater internality-focused HLoC profile was related to lower levels of disability in patients with rheumatoid arthritis and fibromyalgia syndrome, again demonstrating the relationship between HLoC status and outcomes related to pain.^29^ While the Internality model in this current study is generally supported, the results for HLoC Externality model were surprising because no such moderation effects were found. One study considered the relationship between HLoC Internality and Externality with pain intensity and fear avoidance in a cohort of workers with occupational back pain.^47^ They report a generally stronger positive association for back pain intensity for those with high external beliefs (powerful others component) compared to those with low internal beliefs. This may offer some explanation why no moderation effect was found within the Externality model. It may be that because those with high Externality have an already stronger relationship with pain, there was less variance for a moderation effect to occur. Examination of the beta coefficients in our model does show a generally stronger association between pain/pain interference and depressive symptoms in the Externality model, which may offer some explanation to the reported lack of effect. To investigate this further, the difference in mean scores for all variables within the model between high and low groups for both Internality and Externality was inspected. Results show a greater difference in all variables for Internality between the Internality categories (eg, greater Student’s *t*-test scores), compared to the groups within Externality (data not shown); this potentially may have enabled a greater moderation effect to occur for Internality.

### Strengths and limitations

Use of a novel approach is a key strength of this study. This is the first study to test the moderation effects of HLoC in those with LBP, aligned to a theoretical model, which gives greater strength to the interpretation of results. The use of novel statistical techniques (eg, SEM path analysis using pairwise comparison tests) is an additional strength, as this model can indicate not only the presence of a moderation effect (eg, similar to statistical interaction terms or stratification) but also where on a pathway moderation occurs, which may be clinically useful. Added to these strengths is the combination of two LBP populations from a primary care setting which may be more representative of the general population, given that upwards of 95% of the UK population are registered with General Practice.^60^ This is in comparison to previous LBP studies on HLoC which recruited participants from pain management clinics,^61^ or work insurance claim registers.^47^ Another positive aspect of this study is that while the original objectives of these two cohorts differ (creation of trajectories in BaRNS,^33^ and identification of illness perceptions in BeBack),^34^ both cohorts contained the same measures to enable successful combination for this study, which provided adequate sample size and power to test the hypotheses. A limitation of this research is the cross-sectional design, as there is no way of knowing whether depression influences the reports of pain intensity and pain interference, or whether disability and bothersomeness influence depressive symptoms (ie, the reverse direction of our model). However, this issue is minimized for our analysis of moderation, because the moderated effect is less influenced by model direction (ie, HLoC is not an integral part of the structure or direction of the model). The 11 item measure of HLoC used in this study is the forerunner to the more established multidimensional HLoC scale,^62^ which separates the Externality scale into components of “chance” and “powerful others” and adds a “God” scale. Further analysis using these dimensions may have revealed more informative results. Our cohort also consists of those who had reported LBP previously (ie, part of a long-term follow-up study) and it is possible, that through time, the relationship between pain, disability, and HLoC may have changed (ie, those who have continual or persistent pain over time may have perceived less control). However, this study purposefully chose moderation based on the premise that HLoC is a stable characteristic within the individual,^63^ and in supporting this view, research has shown the relative stability of HLoC constructs over time in those with LBP.^31^ Further longitudinal prospective research is needed to give clarity to these issues.

### Clinical relevance

The key finding is that those individuals who have a low sense of control over their health (low Internality) are much more likely to report depressive symptoms resulting from the pain and pain interference they experience. The size of effect shows an almost doubling of the association between pain and depression for those with low Internality. This suggests that HLoC Internality may be a useful prognostic marker for poor outcomes such as depression in those with LBP; however, it may also be true that a person’s depression has influenced their sense of HLoC Internality and further longitudinal research is required to test the development aspects of the model. While HLoC is considered a stable characteristic of the individual, there is evidence to suggest change can be achieved. For example, an intervention targeting HLoC in those with diabetes demonstrated improved outcomes.^64^ Success has also been shown in pain populations; for example, a study reported that a targeted cognitive intervention increased processing and reasoning to enhance perceived control of health (ie, raised HLoC Internality),^65^ and another study showed that a multidisciplinary management approach can enhance Internal HLoC beliefs and minimize External HLoC beliefs by increasing self-efficacy, which led to a reduction in the level of impairment for those with chronic pain.^66^ If clinicians are able to establish a patient’s HLoC status during their initial consultation of LBP, they may be able to utilize this information to recognize potential negative illness perceptions, and could apply positive management strategies to address such beliefs.

In conclusion, this study has shown that low Internal HLoC significantly moderates the pain to depression pathway within individuals with LBP. The findings suggest that HLoC status could be an effective prognostic marker, and further prospective research is needed to test this hypothesis. HLoC may also be amenable to change and the clinician may wish to tailor their clinical management strategies to address negative health locus beliefs in individuals who suffer with LBP.

## Figures and Tables

**Table 1:** Characteristics of study participants

Characteristics (n=637)		Mean (SD)	Interquartile range	Minimum to maximum range	Percentage
Demographics	Age (years)	55.8 (8.8)	13.0	28–69	
	Gender (female)				62.3%
Disability	RMDQ	5.7 (6.1)	8.0	0–24	
Pain	Pain intensity	2.8 (2.7)	3.8	0–10	
	Pain interference	3.1 (2.9)	5.0	0–10	
	Bothersomeness	2.45 (1.2)	2.0	1–5	
Psychological	HADS depression score	4.9 (4.0)	5	0–20	
Health behavior	HLoC Internality score	16.4 (4.3)	6	5–30	
	HLoC Externality score	19.0 (5.9)	8	6–36	

**Abbreviations:** RMDQ, Roland–Morris Disability Questionnaire; HLoC, health locus of control; HADS, Hospital Anxiety and Depression Scale; SD, standard deviation.

**Table 2:** Comparison of low Internality and high Internality pathway standardized beta coefficients and moderation effects

HLoC group	Pathway tested	β (95% CI)#	Critical ratio test
Low Internality	Pain/pain interference to depressive symptoms	0.48 (0.35, 0.62)*	2.41*
	Depressive symptoms to disability	1.62 (1.27, 2.21)*	1.46
	Depressive symptoms to bothersomeness	1.96 (1.52, 2.73)*	1.70
High Internality	Pain/pain interference to depressive symptoms	0.28 (0.18, 0.40)*	–
	Depressive symptoms to disability	2.81 (1.99, 4.06)*	–
	Depressive symptoms to bothersomeness	3.23 (2.26, 4.74)*	–

**Notes:**

* *p*<0.05, β – standardized beta.

# Bias corrected 95% CI.

**Abbreviations:** CI, confidence interval; HLoC, health locus of control.

**Table 3:** Comparison of low Externality and high Externality pathway standardized beta coefficients and moderation effects

HLoC group	Pathway tested	β (95% CI)#	Critical ratio test
Low Externality	Pain/pain interference to depressive symptoms	0.41 (0.23, 0.57)*	0.46
	Depressive symptoms to disability	1.89 (1.41, 3.05)*	0.52
	Depressive symptoms to bothersomeness	2.16 (1.59, 3.73)*	0.25
High Externality	Pain/pain interference to depressive symptoms	0.42 (0.27, 0.55)*	–
	Depressive symptoms to disability	1.89 (1.46, 2.79)*	–
	Depressive symptoms to bothersomeness	2.21 (1.67, 3.45)*	–

**Notes:**

* *p*<0.05, β – standardized beta.

# Bias corrected 95% CIs.

**Abbreviations:** CI, confidence interval; HLoC, health locus of control.
